# Decreased functional activity of multidrug resistance protein in primary colorectal cancer

**DOI:** 10.1186/s13000-015-0264-6

**Published:** 2015-04-16

**Authors:** Tamás Micsik, András Lőrincz, Tamás Mersich, Zsolt Baranyai, István Besznyák, Kristóf Dede, Attila Zaránd, Ferenc Jakab, László Krecsák Szöllösi, György Kéri, Richard Schwab, István Peták

**Affiliations:** 1st Department of Pathology and Experimental Cancer Research, Semmelweis University, Üllői út 26, H-1085 Budapest, Hungary; Rational Drug Design Laboratories, Cooperative Research Center, Semmelweis University, Üllői út 26, H-1085 Budapest, Hungary; Department of Surgery and Vascular Surgery, Uzsoki Teaching Hospital, Uzsoki street 29, H-1145 Budapest, Hungary; Tumorgenetika Human Biospecimen Collection and Research, Kerékgyártó u. 36-38, H-1147 Budapest, Hungary; Podmaniczky u. 63, H-1063 Budapest, Hungary; MTA-SE Pathobiochemistry Research Group, Department of Medical Chemistry, Semmelweis University, Tűzoltó utca 37-47, H-1094 Budapest, Hungary; KPS Medical Biotechnology and Healthcare Services Ltd., Retek utca. 34, H-1022 Budapest, Hungary; Hungarian Academy of Sciences,Research Centre of Natural Sciences, Institute of Molecular Pharmacology, Department of Biological Nanochemistry, Pusztaszeri út 59-67, 1025 Budapest, Hungary; 1st Department of Surgery, Semmelweis University, Üllői út 78, 1082 Budapest, Hungary

## Abstract

**Background:**

The ATP-Binding Cassette (ABC)-transporter MultiDrug Resistance Protein 1 (MDR1) and Multidrug Resistance Related Protein 1 (MRP1) are expressed on the surface of enterocytes, which has led to the belief that these high capacity transporters are responsible for modulating chemosensitvity of colorectal cancer. Several immunohistochemistry and reverse transcription polymerase chain reaction (RT-PCR) studies have provided controversial results in regards to the expression levels of these two ABC-transporters in colorectal cancer. Our study was designed to determine the yet uninvestigated functional activity of MDR1 and MRP1 transporters in normal human enterocytes compared to colorectal cancer cells from surgical biopsies.

**Methods:**

100 colorectal cancer and 28 adjacent healthy mucosa samples were obtained by intraoperative surgical sampling. Activity of MDR1 and MRP1 of viable epithelial and cancer cells were determined separately with the modified calcein-assay for multidrug resistance activity and sufficient data of 73 cancer and 11 healthy mucosa was analyzed statistically.

**Results:**

Significantly decreased mean MDR1 activity was found in primary colorectal cancer samples compared to normal mucosa, while mean MRP1 activity showed no significant change. Functional activity was not affected by gender, age, stage or grade and localization of the tumor.

**Conclusion:**

We found lower MDR activity in cancer cells versus adjacent, apparently, healthy control tissue, thus, contrary to general belief, MDR activity seems not to play a major role in primary drug resistance, but might rather explain preferential/selective activity of Irinotecan and/or Oxaliplatin. Still, this picture might be more complex since chemotherapy by itself might alter MDR activity, and furthermore, today limited data is available about MDR activity of cancer stem cells in colorectal cancers.

**Virtual slides:**

The virtual slide(s) for this article can be found here: http://www.diagnosticpathology.diagnomx.eu/vs/1675739129145824

## Background

ABC-(ATP-Binding Cassette) transporters are transmembrane proteins expressed in the physiological barriers of the human body pumping out a high diversity of substrates (toxins, chemotherapeutics, medications, bile acids etc) from the cells and thus have important role in the detoxification of our body against xenobiotics. Activation of the same MDR-transporters of cancer cells can cause multidrug resistant phenomenon interfering with response to chemotherapy [1].

The clinically most important ABC-transporters are the MDR1 (MultiDrug Resistance protein 1, P-glycoprotein-170) having prognostic role in acute myeloid leukemia [2], sarcomas [3,4] and gallbladder carcinoma [5]; and MRP1 (Multidrug resistant Associated/Related Protein 1), which has prognostic relevance in neuroblastoma [6], hepatocellular carcinoma [7] and in non small cell lung cancer [8].

Based on the high expression of the ABC-transporters along the gastrointestinal tract [9] and the intrinsic low response rate of GI cancers to chemotherapy, colorectal cancer was thought to be chemoresistant due to MDR-proteins [10-13], but later studies have not justified this theory [14-19]. The main therapy of colorectal cancer is the surgical resection of the tumor in combination with chemo (radio) therapy. Chemotherapeutic regimens were initially based on 5-fluorouracil (5FU - neither MDR1 nor MRP1 substrate), but recently Irinotecan (MDR1 substrate) and Oxaliplatin (MRP1 substrate) were also introduced in combination with monoclonal antibodies and resulted in better response-rate and survival rate even in metastatic cases [20,21].

As newer chemotherapeutics raised the possible role of MDR-transporters’ activity in response to therapy, we decided to study the functional activity of MDR1 and MRP1-proteins in freshly isolated viable colon carcinoma cells and normal epithelial cells with the modified calcein assay [22]. In our study of 73 cancer and 11 normal mucosa we found that MDR1 functional activity of colorectal cancer cells was decreased compared to normal enterocytes, while functional activity of MRP1 didn’t change significantly.

## Methods

### Patient samples

Clinical samples were obtained after approval by the national and local Ethical Committees at the Department of Surgery and Vascular Surgery of the Uzsoki Teaching Hospital, Budapest. All patients were enrolled after written consent and altogether 100 samples of primary colorectal cancer and 28 normal mucosal samples were taken into RPMI 1640 (11875-093, Gibco Invitrogen, Grand Island, NY) medium within 30 minutes after devascularization. Colon cases (n = 44) were chemotherapy naïve, while rectal cases (n = 29) received previous chemo-radiotherapy. The samples were stored at 4°C until being processed. Clinicopathological characteristics of the cases involved in the statistical analysis are shown in Table 1.

**Table 1:** **Basic clinicopathological characteristics of studied primary ColoRectal Cancer cases included in statistical analysis**

	**CRC = 73**	**Average age**	**Mucosa = 11**
Males	39	65,2	7
Females	34	68,6	4
Right colon:20	Coecum:12	Ascendens:8	
Left colon:53	Descendens:7	Sigma:17	Rectum:29
Grade	Grade I:9	Grade II:53	Grade III:11
T1:5	T2:20	T3:36	T4:12
TNM Stage I:22	TNM Stage II:21	TNM Stage III:11	TNM Stage IV:19
T1N0M0:4	T1N0M1:1	T2N0M0:18	T2N1M0:2
T3N0M0:18	T3N0M1:3	T3N1M0:9	T3N1M1:6
T4N0M0:3	T4N1M1:9		

pTNM version 6 was used during data collection. (CRC: Colorectal Cancer; pTNM: pathological TNM-stratifictaion).

### Modified Calcein assay for solid tumors

The samples were processed with the modified calcein assay [22]. Surgical samples were cut into small pieces, washed in HBSS buffer (14025-092, GIBCO Invitrogen, Csertex, Budapest, Hungary) then incubated in 1 ml of 4 mg/ml collagenase (LS004212, Worthington collagenase type IV, Worthington Biochemical Corporation, NJ) while continuously mixing for 10 min at 37°C. The reaction was stopped by adding 200 μl 10% FBS (Foetal Bovine Serum – F-2442, Sigma-Aldrich, Budapest, Hungary). After filtering and washing the samples in HBSS, 600 μls of the yielded single-cell suspension were aliquoted into seven tubes.

The dual MDR1 and MRP1 inhibitor Verapamil (V4629, Sigma-Aldrich, Budapest, Hungary) was diluted in HBSS to 250 μM and 200 μl was added to three vials. The MRP1 inhibitor MK571 (340-021-M005, Alexis Biochemicals, Bio-Marker, Gödöllö, Hungary) was diluted in HBSS to 50 μM and 200 μl was added to another two vials. 200 μl HBSS buffer was added to the remaining two control vials. All samples were mixed gently, but thoroughly and subsequently 200 μl of 50 nM HBSS-diluted calcein-AM (C3100, Molecular Probes, Bio-Science, Budapest, Hungary) was added to each sample and incubated for exactly 10 minutes at 37°C. Samples were then rapidly chilled on ice for 5 minutes and spun down. Supernatant was discarded and cells were resuspended in 200 μl HBSS containing 2 μg/ml 7-AAD (AminoActiomycinD – A9400, Sigma-Aldrich, Budapest, Hungary). 1 μg of isotype negative control mouse IgG1 (X093101-2, Dako-Frank Diagnosztika Kft., Budapest, Hungary) was added to one Verapamil treated sample and 1 μg of anti-BerEP4 mouse IgG1 antibody (M080401-2, Dako, Budapest, Hungary) to the other six samples. Subsequently, 0,5 μg of secondary Cy5 conjugated goat anti-mouse IgG antibody (115-175-003, Jackson Immuno Research, Izinta, Budapest, Hungary) was added to each sample and incubated in dark at room temperature for 30 minutes. Samples were spun down and resuspended in 200 μl HBSS containing 1 μg/ml 7-AAD and kept on ice until measurement.

**Figure 1:**
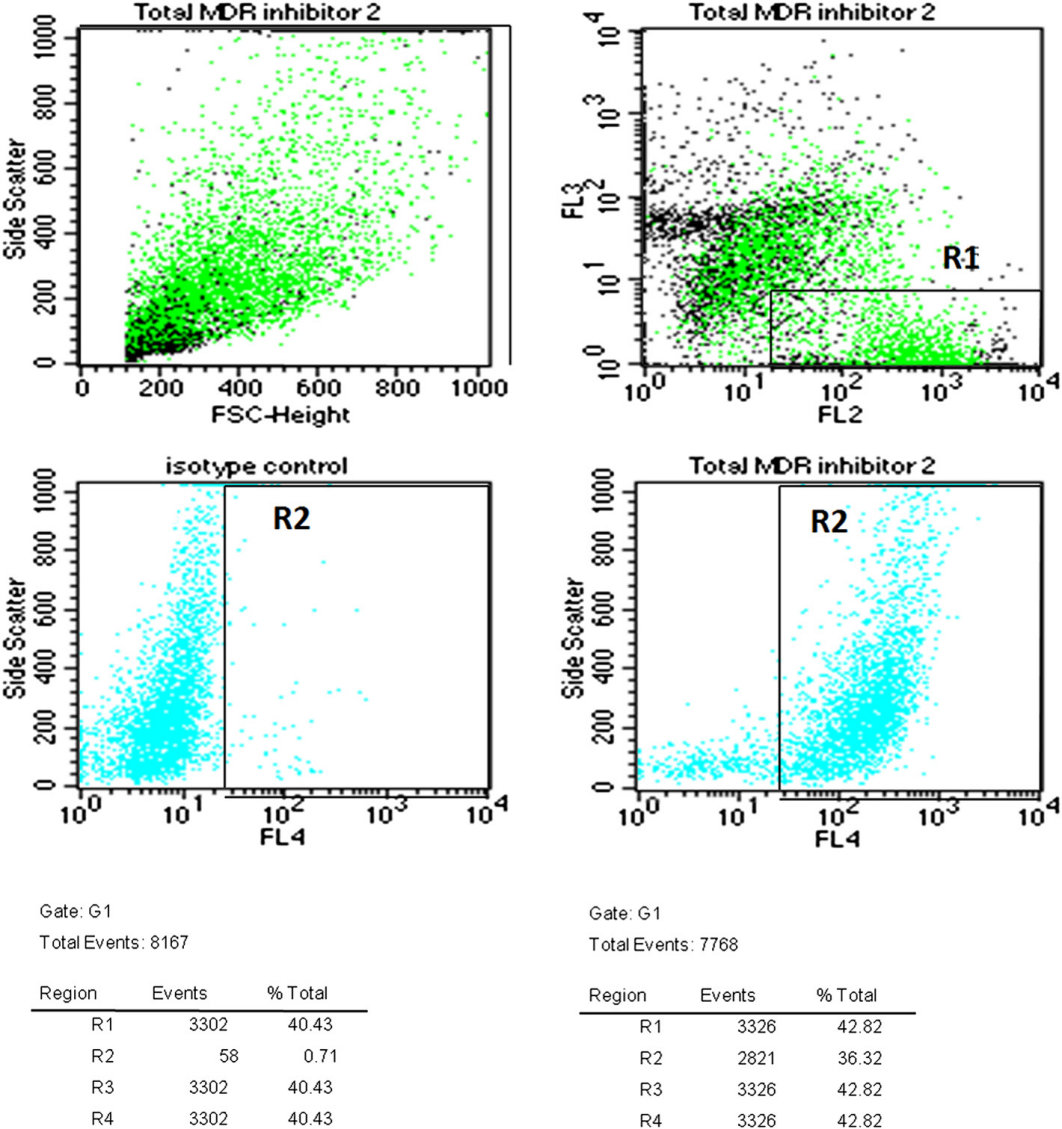
Description of the gating procedure of the viable epithelial cells. FSC-SSC dot-plot was used for the visualisation of the various cell populations in this sample of a colorectal cancer. The viable cells (which were negative for 7AAD and positive for calcein) were selected with R1 gate on FL2 (calcein fluorescence)-FL3 (7AAD signal) dot-plot. R1 was further analyzed for gating out viable epithelial cells with R2 on two parallel FL4-SSC dot-plots of isotype control (middle, left) and BerEP4 antibody binding (middle, right) upon high FL4-BerEP4-positivity. Lower tables show number of cells in each gate. Only cells within R1 and R2 gates were used for the determination of the functional activity of MDR1 and MRP1 transporters.

**Figure 2:**
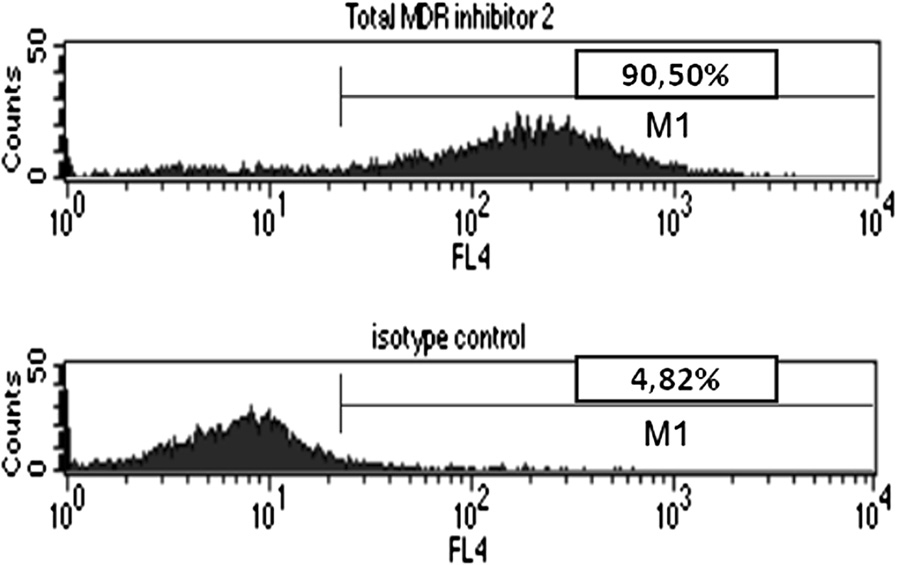
Determining the percentage of viable epithelial cells. The percentage of epithelial cells among viable cells was calculated on FL4 histograms with M1 upon their positivity with BerEp4 (upper graph) compared to isotype control (lower graph). Here 90,50% - 4,82% = 85,68% of viable cells proved to be BerEP4 positive viable epithelial cells. Graphs are representing similar data as the two middle graphs in Figure 1, but numerical analysis worked better with this representation.

**Figure 3:**
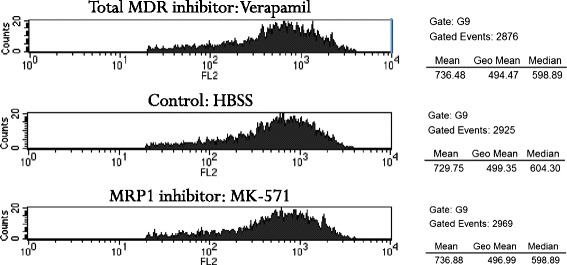
Calculating the MAF-values of viable epithelial cells. The functional activity of MDR1 and MRP1 transporters are calculated with FL2 (calcein fluorescence) histograms showing the impact of the various MDR inhibitors on the mean fluorescence intensity shift. The total inhibitor (Verapamil blocks MDR1 and MRP1) histogram and the MRP1 inhibitor (MK-571) histogram are compared to the control histogram (HBSS, in the middle). In this sample no shift could have been seen with either inhibitor indicating low MDR1 and MRP1 functional activity. Mathematic formula was the following: MAF_Total_ = 100 × (F_Verapamil_ – F_HBSS_)/F_Verapamil_; MAF_MRP1_ = 100 × (F_MK571_ – F_HBSS_)/F_Verapamil_; MAF_MDR1_ = MAF_Total_ – MAF_MRP1_. Where F means the mean Calcein-fluorescence values determined on FL2 in the different samples individually. Here F_Verapamil (Total MDR Inhibitor)_ = 736, F_HBSS (Control)_ = 730, F_MK571 (MRP1 inhibitor)_ = 737; which equals MAF_Total_ = 1, MAF_MRP1_ = 1, MAF_MDR1_ = 0

Flow cytometric analysis was performed on Becton Dickinson FACSCalibur flow cytometer as shown in Figures 1, 2 and 3. Calcein signal was detected on FL-2 instead of the usual FL-1 for better electronic compensation (for details see [22]). Viable cells were gated and selected based on the positive calcein (FL-2) and negative 7-AAD (FL-3) signal of those and further analyzed on the FL4 (BerEp4 signal) and SSC diagram (Figure 1). BerEP4 negative cells were excluded with parallel gating of IgG negative control and BerEp4 samples (Figure 2) and calcein signal shifts of BerEp4 and calcein positive, but 7AAD-negative cells were detected in each parallel samples (Figure 3).

Our assay used the Calcein-AM as a known substrate for both examined transporters. With the two transporter inhibitors (Verapamil for both MDR1 and MRP1 and MK571 only for the MRP1) the assay calculates the individual functional activity of both transporters as MAF-values (Multidrug Activity Factor) of the given sample. MAF values were calculated from the means of calcein fluorescence signals detected with the control HBSS and with the two inhibitors according to the mathematic formula: MAF_Total_ = 100 × (F_Verapamil_ − F_HBSS_)/F_Verapamil;_ MAF_MRP1_ = 100 × (F_MK571_ − F_HBSS_)/F_Verapamil;_ MAF_MDR1_ = MAF_Total_ – MAF_MRP1,_ where F denotes the mean Calcein-fluorescence value determined as the average of the two parallel FL2 signals in the different samples. Samples with highly active MDR1 and MRP1 functional activity give MAF around 20-40, or higher, while samples without significant activity would show values of 0-5. Negative values are probable signs of other active transporters than MDR1 or MRP1.

The absolute number of all cells and viable cells, and the absolute number and percentage of viable epithelial cells were determined in each sample. The MAF-values were not affected by the cell number or cell viability or elapsed time from surgical sampling. Experiments yielding too few viable epithelial cells (under 100) were excluded, thus altogether 11 normal and 73 tumor samples could have been included in the statistical analysis.

Results were tested for normal distribution using the Kolmogorov-Smirnov test with Lilliefors significance correction. Homogeneity of variances was evaluated using the Levene test. For analysis of the variables that slightly derived from normal distribution and homoscedasticity, the two-sample unequal-variance Student’s *t*-test was used [23], while other parameters were compared by equal variance *t*-test. Data are presented as mean +/- SD if normal, and median and inter-quartile range if non-normal. Data analysis was performed using the SPSS 17.0 software (SPSS Inc., Chicago, USA).

## Results

**Figure 4:**
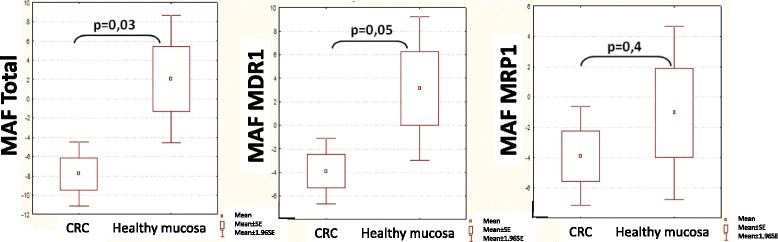
Categorized Box & Whisker plots showing the mean MAF-values and their standard errors in the different groups. CRC stands for the colorectal cancer samples, while healthy mucosa means the normal adjacent mucosa taken from the resection ends. There is a significant decrease in MAF_Total_ and MAF_MDR1_ values, while MAF_MRP1_ remained practically unchanged.

There was a significant decrease (p = 0.03) in the MAF_Total_ values of colorectal cancer cases (MAF: -7.80 ± 14.43) compared to the adjacent, apparently normal mucosa (MAF: 2.08 ± 11.17). This decrease was mainly due to the significant (p = 0.05) decrease in MAF_MDR1_ of colorectal cancer cases (MAF:-3,9 ± 12,12) compared to normal mucosa (MAF: 3,13 ± 10,30), while MAF_MRP1_ values did not differ significantly (p = 0.4), -3.9 ± 14,23 in cancer versus -1,05 ± 9,69 in normal mucosa (Figure 4). The highest MAF-values were detected among the healthy samples and the lowest MAF-values among the cancer samples.

The percentage of epithelial cells and viable cells, and also the heterogeneity and absolute calcein fluorescence values of cells were not significantly different between the control and tumor groups, meaning that the two groups were not different in their main characteristics. ANOVA analysis of tumor localization, left or right sided tumors, TNM stage, grade, age and gender or previous chemo-radiotherapy showed minor, not significant differences in MAF values of MDR1 and/or MRP1.

## Discussion

Because of their high expression in normal gastrointestinal epithelium, MDR1 and MRP1 proteins were considered to be also highly active in colorectal cancers [12]. Early investigations showed higher mRNA and expression levels of MDR1 in colorectal cancers [24], and carcinogenesis [10,11,13], but immunocytochemical [14] and immunoblotting studies [15] have found decreased MDR1 expression in tumor cells compared to the maintained expression in normal mucosa. Furthermore, discrepancy was described between MDR1 mRNA levels and MDR-phenomenon, concluding that phosphorylation status and localisation of MDR1 showed the strongest correlation with functionality [16]. Some studies raised the prognostic role of P-Gp in CRC [25,26], however recent studies found no impact of MDR-expression on survival [13,27,28], not even the largest study with 102 cases [29].

More studies found decreased MRP1 expression with no impact on survival in gastrointestinal tract carcinomas [30,31]. Significant association of elevated MRP2-expression (and not MDR1 or MRP1!) was found in cisplatin resistance [32]. Constitutive MRP1 expression was described in a study of primary and metastatic colorectal cancers, whereas the same study found elevated MRP1 expression in metastatic cases which underwent chemotherapy [33], underlining the impact of previous chemotherapy on the presence and function of these transporters in cancer cells.

The discrepancy between expression study results and clinical findings could be partly resolved with functional studies, which measure the direct transport activity of these pumps regardless of expression or any posttranslational modifications. On the other hand, there are other mechanisms possibly resulting in multidrug resistance phenomenon, so MDR1 and MRP1 proteins might not play key-role in colorectal malignancies [34-37]. Recent research of cancer stem cells (CSCs) in CRC brought the renaissance of the MDR-phenomenon, since the tumor repopulating side population of the resistant CSCs are expressing more ABC-transporters, especially ABCG2 (MXR, BCRP) [38-40].

The modified calcein assay for solid tumors is based on the calcein assay used for prognostication of leukaemia [2] with an added double viability and immunocytochemical staining for selecting living cancer cells. This method has never been used before to investigate large numbers of colorectal samples and up to now very few data is available on activity of these transporters either in healthy or in tumorous colon mucosa [41]. We determined the MDR1 and MRP1 functional activity of normal and cancerous enterocytes in 73 tumor and 11 normal mucosa samples, representing the largest functional study by now. According to our results, multidrug transporter activity of healthy colon mucosa is mainly covered by the functional activity of MDR1 protein, while MRP1 showed lower activity (Figure 4.). The significant MDR1 transporter activity in normal mucosa is in good correlation with previous findings that normal enterocytes express functioning MDR1 transporters. The significant lower mean MAF_Total_-values detected in our colorectal cancer samples were mainly generated by the significant decrease in the mean functional activity of MDR1 transporter, while MAF-MRP1 was practically unchanged.

For now preoperative radio-chemotherapy represents a routine clinical practice in rectal cancers, which means neoadjuvant 50 Gy irradiation combined with 5-FU of the rectal cancers. This treatment had no significant effect on activity of MDR1 and/or MRP1 proteins in the rectal cases (n = 29) involved in our study. Not any other significant differences were found either between the various location of tumors or between left and right sided tumors.

The chemotherapy of colorectal cancer is based on 5-FU, which is neither MDR1 nor MRP1 substrate, but nowadays chemotherapeutic regimen is widening. Irinotecan (MDR1 substrate) and Oxaliplatin (MRP1 substrate) drugs were also involved and succeeded to increase the survival rate of patients. With these newer agents the MDR1 and MRP1 functional activity might influence the response to therapy and possibly also the survival of patients. Functional data determined with our modified calcein-assay protocol could provide more information and insight into the function of MDR-transporters in colorectal diseases. As clinical follow up is in progress, we will be able to study the impact of MDR1 and MRP1 functional activity on the survival of patient in several years.

## Conclusion

In conclusion, our study is the first one to use the modified calcein-assay to determine the MDR1 and MRP1 functional activity of enterocytes and cancer cells from larger numbers of surgical samples of colorectal cancers and healthy mucosa. We measured the MAF_Total_, MAF_MRP1_ and MAF_MDR1_ values of 100 colorectal cancer and 28 normal mucosa samples of which 73 tumor and 11 normal mucosa gave sufficient cells for reliable statistical analysis. We found significant decrease in the MAF_Total_ and MAF_MDR1_ values of colorectal cancer cells compared to the adjacent, apparently normal mucosa. Normal mucosa showed significant MDR1 functional activity, but there was no detectable change in the low MRP1 functional activity between the normal and tumorous mucosa. Univariate and multivariate analysis of tumor localization, TNM stage, grade, age and gender showed no significant impact on multidrug functional activity. Our findings are in good correlation with previous expression studies of MDR1 and MRP1 proteins, which underlined that the expression of MDR1 protein in colorectal cancers is not primarily elevated and probably has no impact on survival of patients. Thus, contrary to general belief MDR activity seems not to play a major role in chemoresistance, but might rather explain preferential/selective activity of Irinotecan and/or Oxaliplatin in CRC. Still, this picture might be to simple, and it is unclear whether chemotherapy by itself might alter this, and furthermore, today a very few is known about MDR activity in CSC. Although, the majority of the chemoresistance of primary CRCs might not be mediated through MDR1 or MRP1 proteins, the combination of predictive molecular diagnostics and MDR diagnostics can potentially further contribute to the advancement of personalized treatment of colorectal cancer patients.

## Abbreviations

5FU: 5-Fluorouracyl
7AAD: 7-AminoActinomycin D
ABC-proteins: ATP-Binding cassette proteins
ABCG2: ATP-binding cassette sub-family G member 2 aka Breast Cancer Resistance Protein (BCRP) or MitoXantrone Resistance Protein (MXR)
BMI: Body mass index
Calcein-AM: Calcein acetoxy methylester
CRC: Colo rectal cancer
CSC: Cancer stem cells
FSC: Forward SCatter
HBSS: Hank’s balanced salt solution
IgG G1: Immunoglobulin G1
MAF: Multidrug activity factor
MDR: Multidrug resistance
MDR1: Multidrug resistance protein 1, also called P-glycoprotein-170
MRP1: Multidrug resistance related protein 1
MDQ Assay: Multidrug quant assay™
RPMI: Developed by Moore et. al. at Roswell Park Memorial Institute
SSC: Side SCatter
